# Photobiomodulation and Wound Healing: Low-Level Laser Therapy at 661 nm in a Scratch Assay Keratinocyte Model

**DOI:** 10.1007/s10439-023-03384-x

**Published:** 2023-10-18

**Authors:** Evdoxia Mathioudaki, Michail Rallis, Konstantinos Politopoulos, Eleni Alexandratou

**Affiliations:** 1 Laboratory of Biomedical Optics and Applied Biophysics, School of Electrical and Computer Engineering, National Technical University of Athens, Zografou Campus, 15780 Athens, Greece; 2 Division of Pharmaceutical Technology, School of Pharmacy, National and Kapodistrian University of Athens, Panepistimioupoli, Zografou Campus, 15771 Athens, Greece

**Keywords:** Wound healing, Keratinocytes, Scratch assay, Low power red laser, Photobiomodulation

## Abstract

This study aims to investigate the effectiveness of low power red light (661 nm) in accelerating the wound healing process of an in vitro scratch assay model of keratinocytes. Furthermore, the study aims to clarify the role of light irradiation parameters, optimize them and gain additional insight into the mechanisms of wound closure as a result of photobiomodulation. Wound healing was studied using scratch assay model of NCTC 2544 keratinocytes. Cells were irradiated with a laser at various power densities and times. Images were acquired at 0, 24, 48 and 72 h following the laser treatment. Cellular proliferation was studied by MTT. ROS were studied at 0 and 24 h by fluorescence microscopy. Image analysis was used to determine the wound closure rates and quantify ROS. The energy range of 0.18–7.2 J/cm^2^ was not phototoxic, increased cell viability and promoted wound healing. Power and irradiation time proved to be more important than energy. The results indicated the existence of two thresholds in both power and irradiation time that need to be overcome to improve wound healing. An increase in ROS production was observed at 0 h only in the group with the lowest healing rate. This early response seemed to block proliferation and finally wound healing. Low level laser light at 661 nm enhanced both proliferation and migration in keratinocytes, providing evidence that it could possibly stimulate wound healing in vivo. The observed results are dependent on irradiance and irradiation time rather than energy dose in total.

## Introduction

The skin is the largest organ of the human body, having multiple roles such as maintaining the hydration levels, regulating the temperature and protecting the body from external factors [1]. A disruption of the skin due to a wound can influence its structure and function, negatively affecting the human body [2]. Wound healing is a non-linear dynamic process, that takes place during four overlapping phases: coagulation/hemostasis, inflammation, proliferation and remodeling [2–4]. Enhancing the wound healing process has been a challenge for many years, with numerous treatments being developed, such as using stem cells, wound dressings, tape removal, electrical stimulation or exposure to laser [5–7].

Photobiomodulation is a healing approach where low-level light exposure to tissues or cells, leads to cell differentiation, proliferation and accelerated wound healing. Non-ionizing photonic energy stimulates photo-biochemical reactions, after absorption of photons by a cellular chomophore, called photoreceptor. In a recent study, Chen et al. reported the existence of two bands in the light spectrum, blue–green and red–infrared that affected wound healing through targeting and stimulation of different photoreceptors [8]. In the red and near-infrared regions of the electromagnetic spectrum (600–1200 nm), laser or LED light devices have been used in photobiomodulation (PBM), phototherapy and photodynamic therapy to induce cell proliferation and the wound healing process [9]. The photoreceptor in this region is within the light-sensitive mitochondrial structures, and more specific it is proposed to be cytochrome-c oxidase (CCO) [9, 10]. As CCO presents two absorption bands, one at the red region (~ 660 nm) and the other in the NIR region (800–850 nm), the most used lasers in PBM are 660 nm, 810 nm, and 850 nm [11]. This region of the light spectrum from red to NIR, is the well-known optical window where light penetration to tissues is maximized. Light with wavelength in the range 600–700 nm is used to treat superficial tissue while longer wavelengths are used to treat deeper—seated tissues [12].

The beneficial effects of photobiomodulation in the red -NIR region appear to be primarily facilitated through the generation of reactive oxygen species (ROS) that increase energy production (ATP) and stimulate enzymes and transcription factors [9, 10]. Several in vitro and in vivo studies have shown the benefits of red light in the wound healing process [13–15]. Nevertheless, the use of photobiomodulation (PBM) has been limited by the lack of standardized protocols, owing to the heterogeneity of experimental or clinical protocols involving cells and animals, and the diverse experimental irradiation parameters employed, such as source type, wavelength, fluence, irradiance, pulse duration, repetition regimen and therapy duration.

Keratinocytes are cells that play a critical role in wound healing. In addition to their role of promoting epithelialization, keratinocytes also play a significant role in immune and inflammatory responses as non-professional immune cells [16].

The aim of this study was to evaluate the effectiveness of red light in promoting wound healing in a scratch model of NCTC 2544 keratinocytes, to elucidate the contribution of light irradiation parameters and finally to optimize them. For this purpose, a diode laser (661 nm) was used and different power and energy rates have been tested for their ability to enhance wound healing by promoting proliferation and/or migration. Furthermore, the generation of ROS was examined to clarify their role in wound healing. Image processing and analysis methods have been used to quantify wound closure rate and ROS production as a result of PBM.

## Materials and Methods

### Cell Culture

NCTC 2544 keratinocytes were a kind gift from Prof. G. Tzanakakis and Prof. D. Nikitovic, Laboratory of Histology – Embryology, Medical School, University of Crete, Greece [17]. They were grown in 75 cm^2^ culture flasks in RPMI 1640 medium (Roswell Park Memorial Institute) (Biosera), enriched with 1% Antibiotic–Antimycotic (Gibco), 0.5% Penicillin-Streptomycin (Gibco), 0.07% Gentamicin solution 1% (Thermo Fisher Scientific) and 10% FBS (Qualified HI/Pen-Str 0.5%, Gibco). Cell cultures were incubated at 37 °C in 5% CO_2_ with 85% humidity. Cells were washed with DPBS 1X (Pan Biotech) and detached with 0.7 mL/25 cm^2^ Trypsin—EDTA 0.05 (Biosera).

### Irradiation Device

Irradiation was performed using a 661 nm diode laser system connected to an optical fiber and a light diffuser (GCSLS-10-1500 m, China Daheng Group) to provide a uniform circular illumination spot. Power output was assessed with a power meter at the cellular level before and after irradiation. The laser spot was centered in the region of interest that is irradiated homogeneously with a power variability of less than 2%.

### Laser Irradiation

Keratinocyte cells were seeded in 96 well plates (0.8 × 10^4^ cells/well) and incubated for 24 h. Then the cells were irradiated with laser light with 40 μL DPBS and the well plate lid was off. The different groups were treated with power output density of 3, 5, 10 and 15 mW/cm^2^ for 1, 2, 3, 5, 6, 8, 10, 13 and 15 min. The irradiation was performed in the dark to avoid any polychromatic light effect. Non-irradiated cells, treated in the same way as the rest, were used as control. All experiments were performed in triplicate.

**Table:** 

	3 mW/cm^2^	5 mW/cm^2^	10 mW/cm^2^	15 mW/cm^2^
1 min	0.18 J/cm^2^	0.3 J/cm^2^	0.6 J/cm^2^	0.9 J/cm^2^
2 min	0.36 J/cm^2^	0.6 J/cm^2^	1.2 J/cm^2^	1.8 J/cm^2^
3 min	0.54 J/cm^2^	0.9 J/cm^2^	1.8 J/cm^2^	2.7 J/cm^2^
5 min	0.9 J/cm^2^	1.5 J/cm^2^	3 J/cm^2^	4.5 J/cm^2^
6 min	1.08 J/cm^2^	1.8 J/cm^2^	3.6 J/cm^2^	5.4 J/cm^2^
8 min	1.44 J/cm^2^	2.4 J/cm^2^	4.8 J/cm^2^	7.2 J/cm^2^
10 min	1.8 J/cm^2^	3 J/cm^2^	6 J/cm^2^	9 J/cm^2^
13 min	2.34 J/cm^2^	3.9 J/cm^2^	7.8 J/cm^2^	11.7 J/cm^2^
15 min	2.7 J/cm^2^	4.5 J/cm^2^	9 J/cm^2^	13.5 J/cm^2^

### Cell Viability Evaluation

Viability was evaluated by MTT {3-(4,5-dimethylthiazol2-yl)− 2,5-diphenyl-2H-tetrazolium bromide, Sigma} assay [18]. Cell medium in each well was replaced with MTT solution at 24 h after the irradiation and cells were incubated for 3 h at 37 °C in 5% CO_2_ with 85% humidity. Then MTT medium was removed and the produced formazan crystals were solubilized with 200 μL DMSO (dimethyl sulfoxide). Absorbance was measured at 570 nm using Microplate Reader (Epoch 2, BioTek Instruments). The relative cell viability was determined as cell survival percentage in comparison to cells that were only treated with complete RPMI medium (controls). All the experiments were performed three times for repeatability.

### Scratch Assay

The keratinocytes were cultured in 12-well plates (38 × 10^4^ cells/well) for 24 h until they form a confluent layer. The scratch was inflicted with a 100 μL sterile pipette tip, across the cell monolayer. The medium was removed and the cells were washed with DPBS to remove the remaining detached cells. Next, 400 μL DPBS was added and irradiation at 661 nm was performed. Afterward, DPBS was removed and replaced with 1 mL RPMI with 1% FBS per well and the plates were put back in the incubator. Images were acquired with an inverted light microscope [Olympus ΙX‐ 81, (Olympus Optical Co., GmbH)] coupled to a CCD camera (XC-30, Olympus} at 0, 24, 48 and 72 h post-irradiation. All the experiments were performed in triplicates. Image acquisition was performed using AnalySIS getIT software (Olympus Soft Imaging Solutions, GmbH). Image J software was used to measure the area of the scratch.

### ROS Production

The production of ROS, as a result of the irradiation with low-power red light, was examined at 0 and 24 h post-irradiation. In order to measure ROS production, chloromethyl-2′,7′-dichlorodihydrofluorescein diacetate (CM-H2DCFDA, Molecular Probes) was used. CM-H2DCFDA was initially dissolved in DMSO and then in RPMI without FBS. Cells (30 × 10^4^ cells/well) were seeded onto coverslips in culture disks for 24 h. After 24 h, coverslips were incubated with CM-H_2_DCFDA (2.7 μM) for 40 min. ROS were monitored right after or 24 h post-irradiation. Coverslips with non-irradiated cells were used as control. After incubation, cells were washed with HBSS (Hank's Balanced Salt Solution, Gibco) and the coverslip was placed in a perfusion chamber allowing live cell imaging. Cells were observed under an epifluorescent upright microscope Olympus BX‐ 50 (Olympus Optical Co., GmbH) using a ×40 objective lens (UPlanFl, N.A. = 0.75, Olympus) coupled to a CCD camera (XC-30, Olympus). The configuration of the filter cube was U‐ MNB excitation BP470‐ 490, dichroic mirror DM500, emission BA515. All the experiments were conducted in the dark. Image analysis and ROS levels were quantified using AnalySIS getIT (Olympus Soft Imaging Solutions, GmbH) software [19].

### Statistical Analysis

Data were analyzed using PRISM software. Shapiro-Wilks test was used to determine the normality of the data. One-way analysis of variance (ANOVA) test was performed to compare all the experimental groups. If the means of the groups are not equal, then multiple comparisons were performed using Bonferroni correction, an adjustment for the big number of comparisons, to explore the differences between experimental groups and control group. Furthermore, groups were categorized in two clusters based on the decision whether or not they differ from control group. In these two new clusters, one-way analysis of variance was performed. Family wise significance and confidence level were set to *P* < 0.05.

## Results

### Cell Viability

**Fig. 1:**
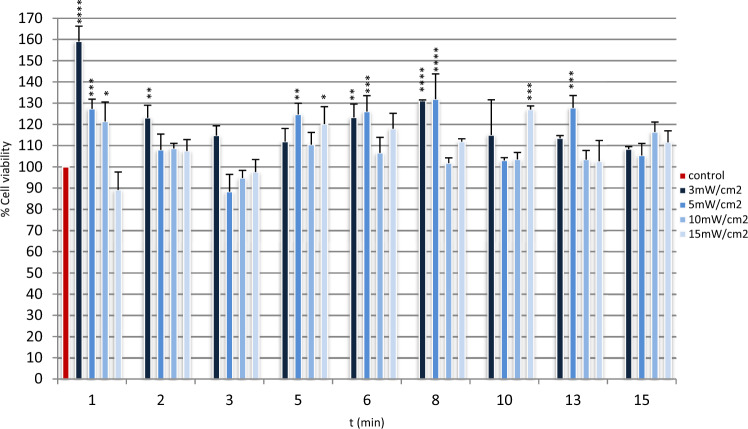
Cell viability results of the different groups, 24 h after irradiation. The data are expressed as the mean of three experiments. The error bars present the standard deviation. Statistically significant differences between irradiated groups and non-irradiated control group are shown as follows: *P < 0.0332, **P < 0.0021, ***P < 0.0002 and ****P < 0.0001

Irradiation of NCTC 2544 keratinocytes with low-power laser light in the red region (661 nm) did not have any toxic effect 24 h after cellular treatment (Fig. 1), for the parameters used in these experiments. Additionally, in many of the experimental groups, cell viability was statistically significantly higher than in the non-irradiated controls indicating an increased cellular proliferation as a result of laser biostimulation.

**Fig. 2:**
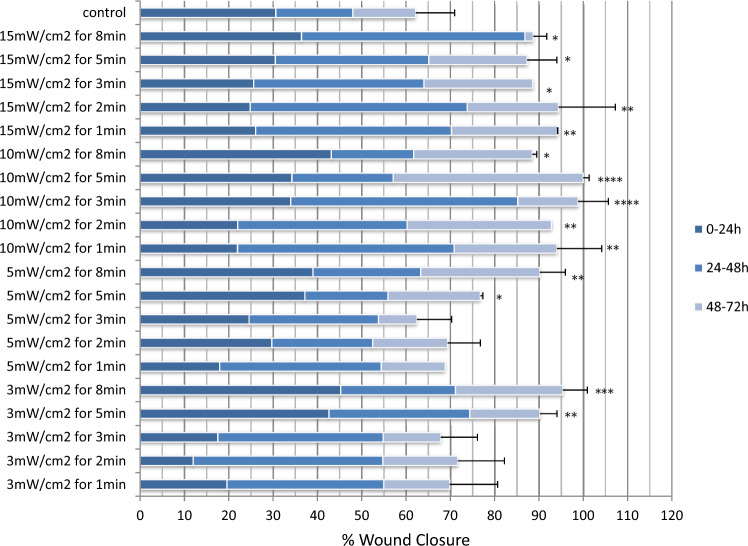
Effect of red light (661 nm) irradiation at NCTC 2544 cells. Scratch wound area was measured 24, 48 and 72 h post-treatment. The error bars present the standard deviation. Statistically significant differences between irradiated groups and non-irradiated control group are shown as follows: *P < 0.0332, **P < 0.0021, ***P < 0.0002 and ****P < 0.0001

As the purpose of the current paper was the study of low-power laser effects on keratinocytes’ role in wound healing, it was decided to test only irradiation times up to 8 min in the scratch assay wound model.

### Evaluation of Wound Healing Assay

After the infliction of the wound, the cells were irradiated with different power outputs densities for 1, 2, 3, 5 or 8 min. Images were acquired 0, 24, 48 and 72 h after the treatment. The wound healing rate was calculated by measuring the area of the “wound” that was not covered with cells after irradiation with the Image J software.

As shown in Fig. 2, 72 h after irradiation, most of the groups presented a greater healing rate than the control one. Multiple comparisons with Bonferroni correction revealed differences between the control group and the most of irradiated ones. More specific, in the groups irradiated with 10 and 15 mW/cm^2^, the observed increase in wound healing rate was statistically significant for all the irradiation times tested (*P* < 0.02 and *P* < 0.04 respectively), while for 3 and 5 mW/cm^2^, only 5 and 8 min revealed a statistically significant wound healing rate (*P* < 0.01 for both cases).

Furthermore, groups were clustered in two categories: the groups that are statistically different from the controls and the groups that are not. One-way ANOVA revealed that there was a statistically significant difference between these two clusters (*P* < 0.05)

**Fig. 3:**
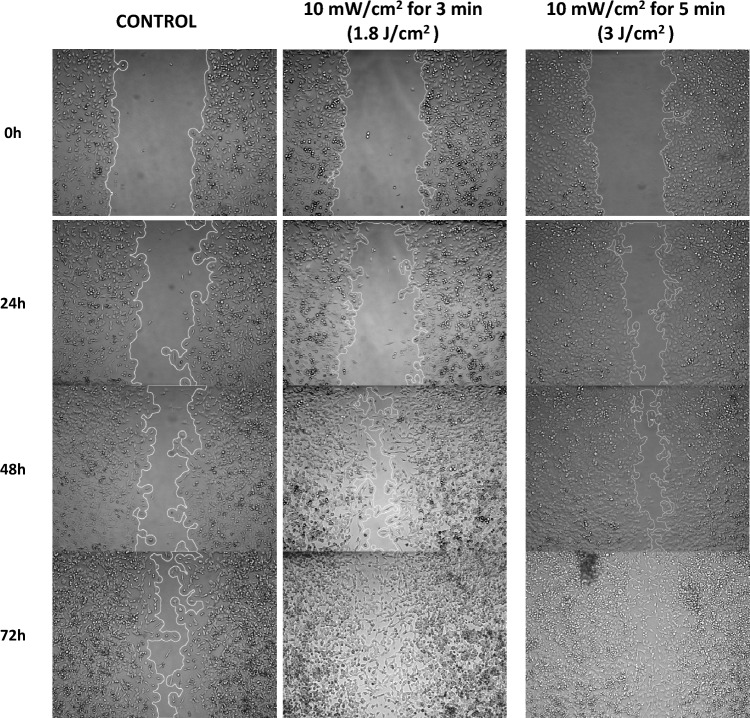
Scratch assay to investigate the wound healing properties of different doses of red light (661 nm) on NCTC 2544 keratinocytes. White lines indicate the area of the scratch at 0, 24, 48 and 72 h after the treatment. Pictures above present the groups with the better healing rate and the non-irradiated control group

In Fig. 3, indicative images for the control and the two outperformed irradiated groups are shown at different time points. Images are processed with Image J and white lines are indicating the wound area.

**Fig. 4:**
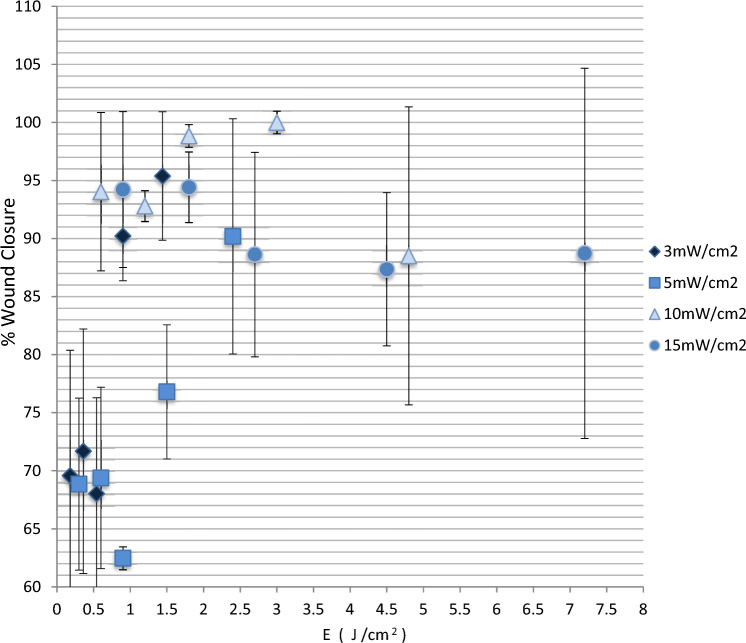
Percentage of wound healing at 72 h after treatment as a function of energy for different values of power density

Moreover, in Fig. 4, wound healing is plotted as a function of fluence rate. As can be seen there, the correlation between wound healing and energy is confusing as smaller energies succeeded in both higher and lower wound healing rates. These findings are also in contradiction with the Bunsen–Roscoe law of reciprocity, which states that if the total energy dose is the same, the photochemical effect should be also the same. But our findings are in agreement with others that also report the failure of the above-mentioned law to describe low-power laser results [12].

**Fig. 5:**
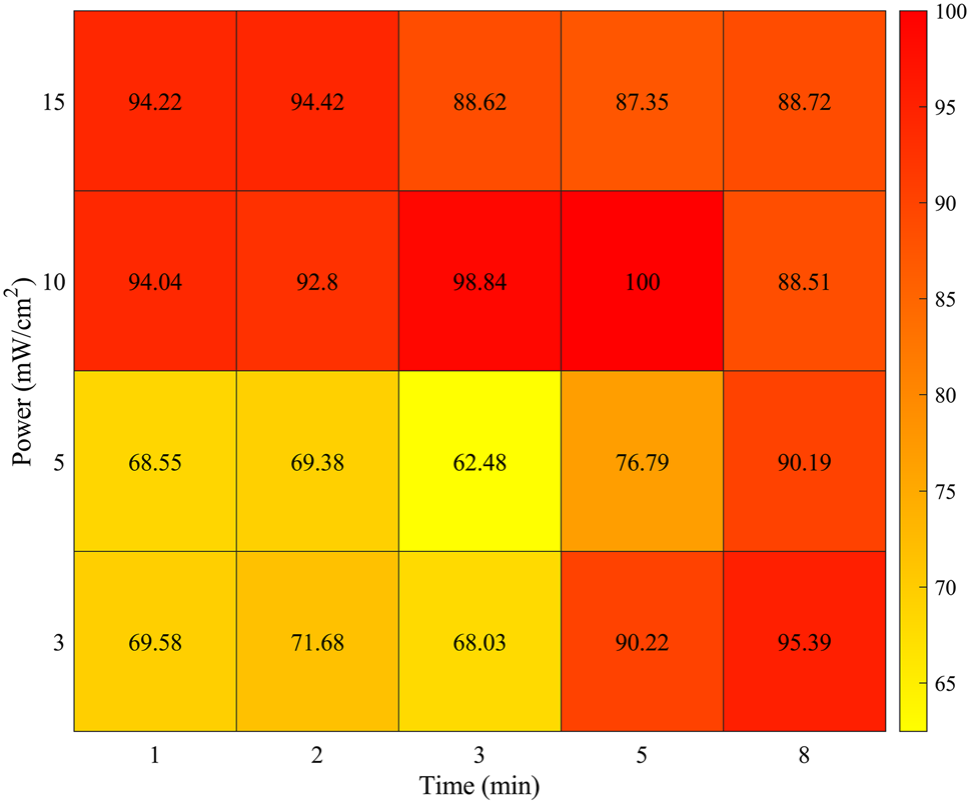
Heatmap of the percentage of wound healing closure in relation to power density and irradiation time

To interpret and further elucidate these findings, power density and irradiation time were used instead of fluence rate. In Fig. 5, a heatmap was created to represent wound healing rates as a function of power density and irradiation time. As illustrated there, two regions provide higher wound healing rates. Firstly, higher power densities are effective even for small irradiation times (1 min), yielding high wound healing rates. But smaller power densities (3 and 5 mW/cm^2^) need longer irradiation times to be more effective and result in higher healing rates. Too short power density and insufficient irradiation time fail to accelerate wound healing. It may be assumed that two thresholds in both power and irradiation time need to be overcome to achieve healing rates that are improved compared to the control.

These results extend our knowledge of the irradiation parameters, indicating that fluence rate is not of special importance while the right combination of power density with time is crucial in achieving higher wound healing rates.

### ROS Production

**Fig. 6:**
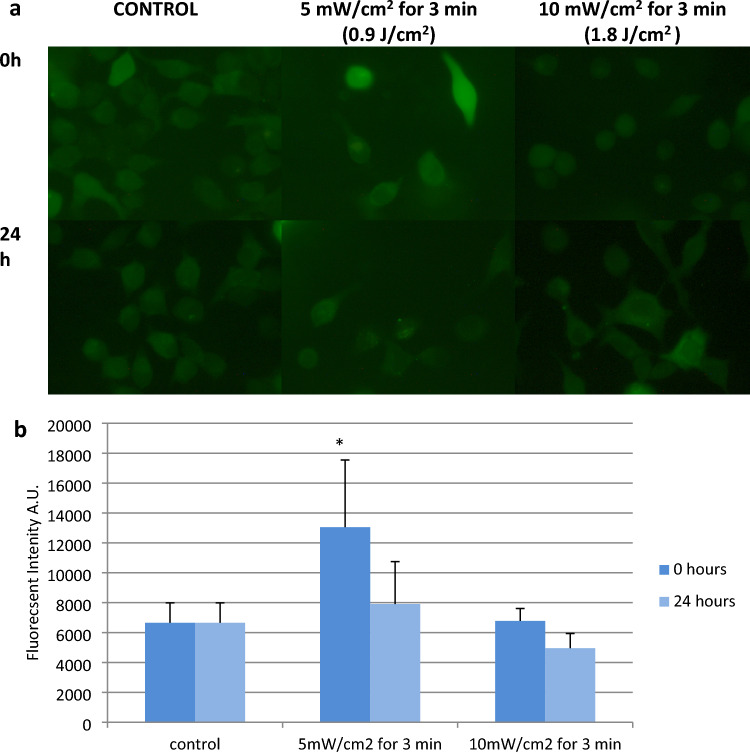
**a** Intracellular ROS levels images 0 and 24 h after the treatment with red light compared to the non-treated control. **b** Quantification of intracellular ROS levels by image processing at 0 and 24 h after the irradiation with red light at 661 nm. The error bars present the standard deviation. *P < 0.05 represents statistically significant differences between experimental and non-irradiated control groups

In order to examine the ROS production from the irradiated cells, three different groups were selected: the control group, the group with the greater closing rate (10 mW/cm^2^, 3 min) and the group with the smallest closing rate (5 mW/cm^2^ for 3 min), comparable to the control group. Image acquisition, evaluation and quantification of ROS production were performed 0 and 24 h after the treatment (Fig. 6a). Immediately after the treatment, the amount of intracellular ROS in the group with the lesser rate is double than that of the control group. The ROS level in the group with the greater healing rate has almost the same quantity of ROS as the control group. However, 24 h after the irradiation, the ROS levels between the control and the 5 mW/cm^2^ for 3 min group are the same but the group with the beneficial dose of 10 mW/cm^2^ for 3 min has even lower ROS levels compared to the other groups as revealed by image analysis (Fig. 6b).

## Discussion

The process of healing is known to be intricate and numerous factors frequently delay its progress. Chronic wounds are a significant health and financial challenge and physicians must find ways to create effective and feasible treatment methods [20]. Therapies that utilize light, specifically red light, have shown potential in accelerating wound healing by promoting the proliferation of different cells such as fibroblasts and keratinocytes [16, 21]. These discoveries propose a hopeful approach to combat delayed wound healing that results in chronic wounds. After an injury, keratinocytes migration begins at 12–24 h. These cells' migration and proliferation are crucial for wound reepithelialization and gap closure [22, 23].

In the current study, NCTC 2544 keratinocytes were used in a scratch assay wound model and were irradiated with laser at 661 nm with various light parameters such as power density, irradiation time and fluence rate in an attempt to study laser photobiomodulation. The research focuses on the evaluation of the effectiveness of low-power red laser to accelerate wound healing, the analysis of the importance of the irradiation parameters, and at last the optimization of them.

Fluence rates from 0.18 to 13.5 J/cm^2^ were tested with different combinations of laser power density and irradiation time. The conditions up to 8 min were examined further in a scratch assay model because neither of them diminished cell viability. The majority of the experimental groups showed greater healing rates than the control. These results suggest that there is a beneficial interval of 0.18–7.2 J/cm^2^ that positively affects keratinocytes’ ability to wound healing. Our findings are consistent with previous results of Topaloglu [9] who used a laser emitting 655 nm at 1, 3, and 5 J/cm^2^ energy and showed induction of proliferation and cell migration on keratinocytes. Further analysis of our data revealed that the crucial parameter in wound healing acceleration through low-power red laser light is not energy but the combination of power density and time. The existence of two thresholds on both power and irradiation time was proposed as successful dosimetry scheme for wound healing acceleration.

In addition, the results of the effects of low-power laser light on cell viability showed increased viability, indicating cellular proliferation, in most of the experimental groups compared to the control. But the group with the highest cellular proliferation did not present the highest wound closure rate and vice versa. As the scratch assay experiments were performed in reduced FBS (1%) compared, it can be concluded that the scratch wound model's closure is influenced by another factor, possibly keratinocyte migration. This hypothesis is further supported by the work of Sutterby [24] and Sperandio [25], suggesting the role of red light on cell migration. Although the two experiments, proliferation and scratch assay, were performed with different FBS supplementations and can not be directly compared, we can assume that in a real wound, low-power laser light will contribute to wound closure by both proliferation and migration.

Further analysis of the time course experiments of wound healing, revealed that on the first day, all wounds had the same healing rate as the control group, indicating no effect of cellular irradiation. An increase in the wound healing rate was evident on the second and third days. Red light biostimulation gave raise to cellular population on the first day and this increased number of cells start to migrate during the second day of the experiment. But if the keratinocytes multiply excessively as in cases of 3 and 5 mW/cm^2^ for 1 and 2 min of irradiation, it can be assumed that they cannot migrate as fast as others resulting in a desirable but not as significant enough wound closure. All these findings suggest that wound healing acceleration requires the balanced contribution of both migration and proliferation.

The fact that power density and time combination are critical for the wound healing effect can also be explained through ROS production mechanisms. PBM is believed that speeds up wound healing by increasing ROS production, which raises ATP levels [9]. It is common knowledge that elevated and sustained ROS levels have been linked to a wide range of adverse effects on cell survival and may diminish wound healing [26, 27]. However, some studies indicate their beneficial role, when ROS production remains under certain levels. Specifically, when low levels of ROS are produced, they can act as a trigger promoting proliferation, wound healing and preventing infections [3, 28, 29].

In the present study, the intracellular production of ROS was examined and quantified at 0 h and 24 h after the irradiation in two different experimental groups: the one that provided the optimal wound healing (10 mW/cm^2^, 3 min) and one from the ineffective groups that did not affect wound healing and presented the same wound healing rate as control (5 mW/cm^2^, 3 min). Interestingly, immediately after the treatment the ineffective group, showed vastly higher intracellular ROS levels (almost double) compared to the other groups. However, 24 h after the irradiation the ROS levels between the control and the other groups are almost the same.

The high ROS production after laser irradiation at the ineffective group can be assumed to act as a harmful agent that can explain the observed responses in wound healing. Irradiation with power density below the threshold caused an increase in the amount of ROS, which would seem to imply detection by cellular ROS sensors as a potentially harmful level of ROS and induction of the expression of antioxidant defenses in order to restore oxidative balance [30]. This very first cellular response to red light, blocked or delayed cellular proliferation and as a consequence wound healing rate. Further experiments, using antioxidants such as polyophenols, ferulic acid or antioxidants already found in skin such as a-tocopherol, ascorbic acid or glutathione should be performed to exploit the exact mechanisms and verify this hypothesis.

In another study conducted also in our laboratory [31], with the same laser source and the same experimental conditions using 3T3 fibroblasts instead of keratinocytes, the wound closure was faster compared to our scratch models. All wounds, including controls, were totally closed within 2 days, whereas in keratinocytes total wound closure was observed in only 2 experimental groups 3 days after irradiation. As far as it concerns the control group, our results concur well with those of Walter et al. that have also reported faster wound closure in fibroblasts than keratinocytes scratch assays [32] without cellular irradiation. But when it comes to laser irradiation, our findings are in contradiction with previous results reported in the literature. Topaloglou et al. have found that 655 nm laser was more successful in keratinocytes wound healing than those of fibroblasts but these results were obtained using a much higher power of 50 mW [9]. Engel et al also reported increased sensitivity of keratinocytes compared to fibroblasts to laser irradiation but they used near-infrared laser at 808 nm [33]. As the two cell types are different in their cellular properties, it is expected to display different cellular properties and responses to various stimuli. So, although comparing the two cell types can’t be considered correct, they are both mentioned here so that it is clear that low power laser light has positive results in all cells that contribute to wound healing either they are in epidermis (keratinocytes) or deeper in dermis (fibroblasts).

The correlation between intracellular ROS levels, cell type and time is also worth mentioning. Keratinocytes presented increased levels of intracellular ROS immediately after the treatment that resulted in no acceleration of wound healing, while fibroblasts showed a delayed increase in intracellular ROS levels 24 h after the treatment that led to a beneficial effect on wound healing. The same observation of different levels of ROS production after the irradiation was noted by Engel [33], after the irradiation of the two types of cells with laser emitting at the near-infrared 808 nm. These results may indicate that different types of cells interact differently with the light providing distinct outcomes that all together contribute to the wound healing process.

In conclusion, the present findings show the impact of low-level laser therapy using a device emitting at 661 nm in a wound scratch model of NCTC 2544 keratinocytes. Image processing and analysis were applied to quantify the wound closure rate and ROS production. The wound healing process was accelerated differently depending on the different laser power and irradiation times that were examined. The study of wound closure after laser irradiation revealed the significant contribution of both proliferation and migration of the cells. ROS production was increased immediately after laser stimulation in the group that showed the slowest wound closure, but interestingly the group that showed the fastest wound closure rate had similar ROS levels as the control group. 24 h after laser stimulation the ROS was at a normal rate in both groups. The results of this research provided a better understanding of the mechanisms and the role of low-level red laser in the acceleration of keratinocytes’ wound healing.
